# Climatic Warming Increases Winter Wheat Yield but Reduces Grain Nitrogen Concentration in East China

**DOI:** 10.1371/journal.pone.0095108

**Published:** 2014-04-15

**Authors:** Yunlu Tian, Chengyan Zheng, Jin Chen, Changqing Chen, Aixing Deng, Zhenwei Song, Baoming Zhang, Weijian Zhang

**Affiliations:** 1 Institute of Applied Ecology, Nanjing Agricultural University, Nanjing, China; 2 Institute of Crop Science, Chinese Academy of Agricultural Sciences/Key Laboratory of Crop Physiology and Ecology, Ministry of Agriculture, Beijing, China; 3 Soil and Fertilizer and Resources and Environmental Institute, Jiangxi Academy of Agricultural Science/Key Laboratory of Crop Ecophysiology and Farming System for the Middle and Lower Reaches of Yangtze River, Ministry of Agriculture, Nanchang, China; North Carolina State University, United States of America

## Abstract

Climatic warming is often predicted to reduce wheat yield and grain quality in China. However, direct evidence is still lacking. We conducted a three-year experiment with a Free Air Temperature Increase (FATI) facility to examine the responses of winter wheat growth and plant N accumulation to a moderate temperature increase of 1.5°C predicted to prevail by 2050 in East China. Three warming treatments (AW: all-day warming; DW: daytime warming; NW: nighttime warming) were applied for an entire growth period. Consistent warming effects on wheat plant were recorded across the experimental years. An increase of ca. 1.5°C in daily, daytime and nighttime mean temperatures shortened the length of pre-anthesis period averagely by 12.7, 8.3 and 10.7 d (*P*<0.05), respectively, but had no significant impact on the length of the post-anthesis period. Warming did not significantly alter the aboveground biomass production, but the grain yield was 16.3, 18.1 and 19.6% (*P*<0.05) higher in the AW, DW and NW plots than the non-warmed plot, respectively. Warming also significantly increased plant N uptake and total biomass N accumulation. However, warming significantly reduced grain N concentrations while increased N concentrations in the leaves and stems. Together, our results demonstrate differential impacts of warming on the depositions of grain starch and protein, highlighting the needs to further understand the mechanisms that underlie warming impacts on plant C and N metabolism in wheat.

## Introduction

Global mean air temperature has increased by about 0.74°C over the last century and is predicted to rise by 2.0 to 5.4°C in this century [1]. Wheat (*Triticum aestivum* L.), the most important staple crop in the world [2], is mainly cultivated in the winter-spring seasons when warming is most likely anticipated [1]. Temperature is a major factor affecting crop development and growth. Thus, even a moderate increase of air temperature is likely to influence wheat yield [3], [4] and grain quality [5]–[7]. China is one of the largest countries of winter wheat production and consumption. The daily mean air temperature has increased by 1.5°C since 1980 in eastern China, the major Chinese cropping area of winter wheat. Moreover, the temperature is expected to increase by an average of 1.2–2.0°C by 2050 in the region [8]. Therefore, it is significant and necessary to examine warming impacts on Chinese winter wheat production [9], [10].

Climatic warming is often predicted to reduce both wheat yield and grain quality in China [8], [11], [12]. Theoretically, warming can shorten the length of wheat growth period, likely resulting in large declines in biomass production and N uptake and accumulation [6], [13]. Warming may also aggravate high temperature stress to wheat growth and grain filling, potentially causing serious decreases in grain number and weight [14], and severely influence grain N accumulation and flour quality [15], [16]. Because annually multiple cropping systems dominate in China, the short growing period and the post-anthesis high temperature are the major constraints on winter wheat production in the country. Thus, warming may aggravate the constraints on Chinese wheat production. On the other hand, temperature increase may directly reduce frost/chilling and indirectly avoid heat injury due to warming-led earlier anthesis [17]–[19]. Since frost/chilling before flowering and high temperature stress after flowering happen often in Chinese winter wheat cropping area, warming may enhance Chinese wheat yield and grain quality. For example, based on historical data analysis and the assumption of rainfall, Xiao *et al*. (2008) predicted that climatic warming might lead to an increase of 3.1% in wheat yield at a low altitude and of 4.0% at a high altitude in China by 2030 [20]. Recently, Sommer *et al*. (2013) also predicted that climate change might mostly benefit wheat production in Central Asia, and the positive effects of climate change might be mainly due to the predicted increase in air temperature [21]. Obviously, there are still some major uncertainties about wheat production under future climate due to the unclear warming impacts on wheat phenology and grain growth in China.

A considerable number of experiments have been conducted to quantitatively evaluate warming impacts on wheat yield and quality during the past decades [2], [6], [22], [23]. However, many of them were conducted under artificial environments [22], [24], [25]. Meanwhile, previous experiments mainly focused on high temperature impacts on starch and protein depositions in wheat grain during post-anthesis phase [26]–[28], and only few studies had been conducted across an entire growing cycle [29], [30]. The impacts of predicted warming on wheat yield and grain quality might have been overestimated [25]. Furthermore, since changes of daily minimum temperature and maximum temperature can affect crop growth differently [1], [31], warming with different diurnal temperature range (DTR) may induce dissimilar impacts on crop production [32], [33]. To date, few have quantitatively assessed wheat responses to air temperature increase with different DTR under field conditions. We, therefore, initiated a 3-yrear field warming experiment in Nanjing, China, in the winter of 2006 with a Free Air Temperature Increase (FATI) facility. Four treatments were designed, i.e., all-day warming (AW), daytime warming (DW), nighttime warming (NW) and non-warmed control (CK). Our objectives were to investigate the responses of winter wheat phenology, biomass production, grain yield and N accumulation to an increase in air temperature under field conditions.

## Materials and Methods

### Site description

The free air temperature increase experiment was conducted from the winter of 2006 to the summer of 2009 at the Experimental Station of Jiangsu Academy of Agricultural Sciences, Nanjing city, China (32°02′N, 118°52′E, 11 m above sea level) (Fig. S1). This station is located in the subtropical monsoon climatic zone. The mean annual temperature is 16.7°C. The annual precipitation is 1050 mm, and the annual sunshine time is 1900 h with 237 frost-free days. The daily mean temperature and the precipitation during winter wheat growing seasons were averagely 11.5°C and 400 mm during 1980–2010, respectively (Fig. S2A). According to field observations, there is no water limitation to winter wheat growth in most years. The times of sunrise and sunset are respectively around 6:05 am and 17:50 pm during wheat growing season. Winter wheat is one of the major crops in East China. The soil is a brunisolic silt loam soil (an Alfisol in USA-ST). Soil sand, silt, and clay are 0.5, 75.3 and 24.2%, and soil organic carbon, total N, total P and total K are 8.2, 2.5, 0.6 and 14.0 g kg^−1^, respectively. The available P and K are 166.2 and 165.0 mg kg^−1^.

### Field warming facility

The free air temperature increase (FATI) facility was constructed based on the design of the warming system located at Great Plain Apiaries, USA [34]. FATI facility has been widely used to study plant responses to warming at ecosystem scale under field conditions [30], [34] and is well suited for determining actual crop responses [23]. According to our previous test finished in 2004–2005, a single 180 cm×20 cm 1500 W infrared heater (Jiangsu Tiande Special Light Source Co., Ltd., China) was suspended 1.5 m above the field ground in each warmed plot (Fig. S1). To eliminate the shading impacts of the heater, a ‘dummy’ heater of the same shape and size was suspended at the same height in each non-warmed control. The distance between adjacent plots was approximately 5 m to avoid heating interference between treatments. The air temperatures on wheat canopy were monitored with an interval of every 20 minutes in each treatment for an entire growing cycle by a digital temperature monitor (ZDR-41, Hangzhou Zheda Electronic Instrument Co., Ltd., China). Three monitors were positioned in the plot centre under the heater or the dummy heater on wheat canopy and field surface and in the 5 cm soil layer, respectively. Automatic monitoring of air and soil temperatures for an entire growth period was conducted in one replicate for each treatment. Meanwhile, the temperatures were also measured manually using thermometer at wheat key stages in all replicate plots, so as to make sure that there were no obvious differences in the warming effects between the replicates. The warming levels and time schedules were automatically controlled according to the ambient air temperature on wheat canopy. And all instruments in this warming system were checked and adjusted yearly before the experiment starting and monthly during the growing seasons according to the automotedly and manually monitoring.

### Experimental design

The field experiment used a randomized block design with three replicates (Fig. S1). Each plot area was 6 m×5 m in size. Four treatments were set up: all-day warming (AW, warmed all day), daytime warming (DW, warmed from 06:00 to 18:00), nighttime warming (NW, warmed from 18:00 to 06:00), and a non-warmed control (CK). The warming treatments were all initiated at the sown date and maintained to the maturity date for an entire growth period of winter wheat. An increase of 1.5°C was set for the experimental warming levels of daily mean temperature, daytime mean temperature and nighttime mean temperature for the AW, DW and NW, respectively. Across the entire growth period, the actual increases were averagely 1.4, 1.3 and 1.5°C of daily mean temperature for the AW, daytime mean temperature for the DW and nighttime mean temperature for the NW, respectively (Table S1). Accordingly, the FATI facility could provide uniform and reliable warming effects within an area of 2 m×2 m in size (Fig. S2B, C), which was similar to the facility located at Great Plain Apiaries, USA [34]. In order to avoid sampling disturbance, this 4 m^2^ area was divided into two equal sup-plots, one for plant and soil sampling at key growing stages, and the others for the determinations of biomass production and grain yield at harvest.

### Crop management

During the experimental years, one winter wheat (*Triticum aestivum* L.) cultivar Yangmai 11, a leading local cultivar with high yield potential, was used. Standard agronomic practices commonly performed for high yield in the area were applied to all plots. Wheat seeds were manually sown in November at a density of 225 plants m^−2^ with a row spacing of 20 cm. Wheat grain was harvested in late May or early June in the following year depending on the maturity date of each treatment (Table 1). The application rates of chemical N, P and K to each plot were 225, 75 and 75 kg ha^−1^, respectively. Half of the N and total P and K were applied as a basal dressing two days prior to sowing. The other of 50% N was applied as side dressings in each plot at early tillering in the beginning of March, based on the wheat development in the non-warmed control. The average precipitation over wheat entire growth period was 365.5 mm across the experimental years (2006–2007: 345.4 mm; 2007–2008: 396.9 mm; 2008–2009: 354.1 mm). Although warming decreased the average surface soil (0–20 cm) moisture by 1.1, 0.37 and 0.41% in the AW, DW and NW plots, respectively, the reductions were not significant compared to that in the non-warmed control (Fig. S2D). Since no obvious soil moisture limitation was found in the field, the fields were not irrigated in the warmed and non-warmed plots during the experimental years.

**Table 1:** Winter wheat phenological dates, aboveground biomass production and grain yield in non-warmed control (CK), all-day warming (AW), daytime warming (DW) and nighttime warming (NW) plots.

Year	Treatment	Date (Year/Month/Day)			Biomass production (g m^−2^)	Grain yield (g m^−2^)
		Sowing	Anthesis	Maturity		
2006–2007	CK	Nov 22	April 15	June 02	1638.2±187.3a	709.3±54.9b
	AW		April 05	May 23	1820.6±109.2a	863.5±20.2a
	DW		April 09	May 26	1943.5±14.5a	833.0±12.7a
	NW		April 06	May 25	1895.4±64.4a	804.7±8.6a
2007–2008	CK	Nov 15	April 14	June 02	1689.6±75.5a	635.9±6.1b
	AW		April 04	May 21	1814.0±60.5a	707.3±10.2a
	DW		April 07	May 25	1765.1±100.4a	729.7±9.7a
	NW		April 05	May 23	1750.9±51.3a	791.1±3.4a
2008–2009	CK	Nov 02	April 10	May 24	1592.8±22.5b	681.2±21.1b
	AW		Mar 23	May 14	1785.6±54.6a	785.6±8.9a
	DW		Mar 29	May 19	2032.5±146.8a	831.4±0.6a
	NW		Mar 27	May 16	2022.8±20.5a	828.7±22.3a

Values are means ±1 SE. Values followed by a different letter are significantly different within the treatments in each year (*P*<0.05).

### Sampling and measurement methods

Since warming promoted wheat plant development, there were differences in the anthesis dates among all treatments. Thus, wheat plant in a particular treatment reached a different phenological stage at same date. Thereby, field sampling and determining were conducted on a plot by plot based on their phenological stage rather than on the same calendar date.

### Plant sampling and determinations

The anthesis date was recorded when 50% of panicles in a plot had flowered, and maturity when most of the panicles in a plot showed complete loss of green color. At the anthesis stage, fifteen plants were sampled from each replicate. The flag leaf and total green leaf area on each plant was measured using a Li-3000 Portable Area Meter (Li-COR, Inc. USA). The mean area of the fifteen plants represented the area of each replicate. At maturity, aboveground plant samples (15 plants) were taken from each replicate. The plants were separated into leaves, stems and panicles. Panicles were further separated into vegetative components and grain. All plant samples were oven-dried to a constant weight at 80°C and weighed. Nitrogen (N) concentrations of leaf, stem and grain were quantified using the Kjeldahl digestion method [35]. Plant samples were placed in digestion tubes and digested with a salt-catalyst-sulfuric acid mixture by heating the tubes in an aluminum block. At maturity, one sup-plot of 1 m^2^ in area without sampling disturbance was harvested to determine the biomass production and grain yield for each replicate.

### Flag leaf photosynthesis

At the anthesis stage, five flag leaves (all from main stems) in each plot were selected at 09:00–11:00 on the clear mornings. The net photosynthetic rates were determined using a Li-Cor 6400 Open Gas Exchange System (Li-COR, Inc. USA) in 2008 and 2009. Air entering the system was drawn out at a 2 m high level, and then passed through 2.5 L volume of buffer before the air re-entered the system. Air flow through the chamber was maintained at 500 µmol s^−1^. To avoid differences in photosynthesis caused by the changing photosynthetic photo flux density (PFD) levels, measurements were all made under a constant PFD at 1600 µmol m^−2^ s^−1^ provided by blue-red light emitting diodes mounted above the leaf cuvette. During the determination, the leaf chambers conditions were controlled automatically, and the CO_2_ level was maintained around 370 µmol mol^−1^. The cuvette air temperature and humidity were kept similar as the natural field conditions of each replicate plot [36].

### Wheat root activity

Samples of the wheat stubble together with the undisturbed soil block (15 cm in length, 20 cm in width and 30 cm in depth) were collected at anthesis. The resulting cavity was refilled with soil blocks taken from a place elsewhere in the same field. The soil blocks with wheat stubble were submerged in water under dark conditions for 20 min and then gently washed in a 4 mm sieve. The roots were clipped off and stored at 4°C waiting for activity determination using the α-naphthyl-amine test [37]. One point five gram roots were placed in a 25-ml test tube before adding 5 ml of 0.1 M Na-phosphate buffer (pH 7.0) and 5 ml of 50 ppm α-naphthylamine. One microliter of solution was taken from the test tube after shaking for 30 min and 3 h, respectively. Then, 1 ml of 1% ρ-aminobenzene sulfonic acid and 1 ml of 0.01% NaNO_2_ were added to the two samples. Absorbance at 510 nm was measured after incubation for 10 min at 30°C. Meanwhile, a control without roots was used to determine the auto-oxidized α-naphthyl-amine content. The α-naphthy-lamine concentrations in the tube after shaking for 30 min and 3 h were determined by comparison with α-naphthyl-amine standard solutions. The oxidized α-naphthyl-amine was calculated as the decrease in the amount of α-naphthyl-amine from 30 min to 3 h. Root activity was expressed as oxidized α-naphthyl-amine (µg) minus auto-oxidized α-naphthyl-amine in the control per gram of fresh root per hour.

### Data analysis

The apparent N remobilization efficiency (NRE) was estimated as the fraction of nitrogen taken up at flowering that was remobilized [38].

NRE =  the difference in N content of vegetative parts between at anthesis and at maturity/N content of the vegetative parts at anthesis ×100.

Data were analyzed with the statistical package SPSS 11.5. Repeated measures analysis of variance (rANOVA) was used to determine significant differences (*P*<0.05). In this experiment, wheat phenological dates, biomass production and grain yield were recorded for three growing seasons (2006–2009), other indexes were determined for two growing seasons (2007–2009).

## Results

### Wheat phenological length and plant productivity

Warming advanced wheat plant development, resulting in significant changes in the phenological dates and the reductions in the length of entire wheat growth period (Table 1). As compared to the non-warmed control, the length of the entire growth period was shortened averagely by 10.7, 6.7 and 8.7 d during the three-year in the AW, DW and NW plots (*P*<0.05), respectively. The main reductions were found in the lengths of pre-anthesis periods (e.g. from sowing to anthesis) respectively by 12.7, 8.3 and 10.7 d in the AW, DW and NW plots (*P*<0.05). However, no significant difference in the length of post-anthesis period (e.g. from anthesis to maturity) was found between the warmed and non-warmed plots.

Warming increased wheat grain yield significantly, while there was no significant increase in aboveground biomass production (Table 1). The grain yield averaged 16.3, 18.1 and 19.6% higher (*P*<0.05) across the three years in the AW, DW and NW plots than the non-warmed control.

### Leaf area, chlorophyll concentration and photosynthesis rate

**Figure 1:**
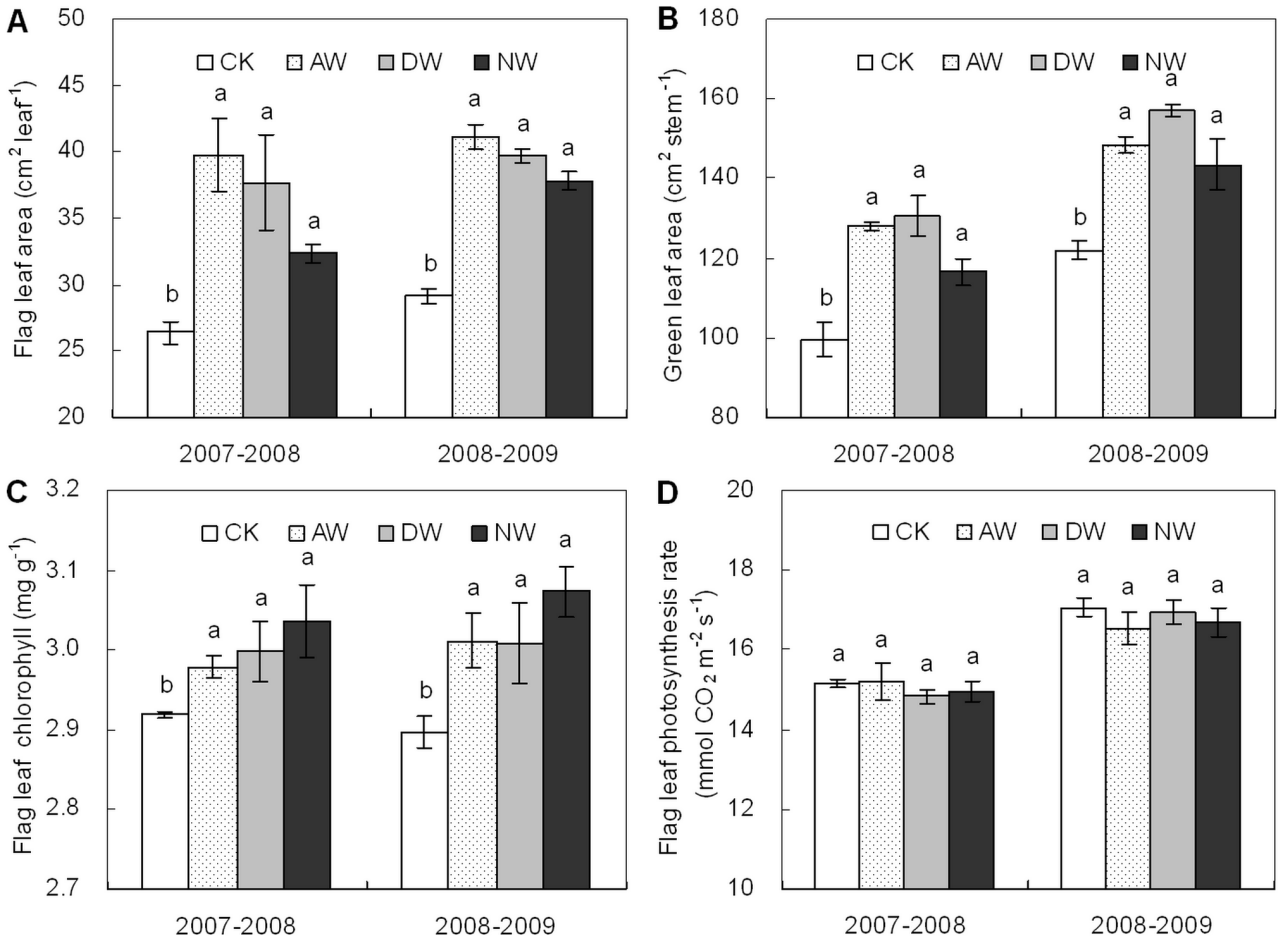
Flag leaf area (A), green leaf area (B), flag leaf chlorophyll a and b concentration (C) and flag leaf photosynthesis rate (D) at anthesis during the 2007–2008 and 2008–2009 growing seasons in the CK, AW, DW and NW plots. The data are not available for the growing season of 2006–2007 since they were not determined in the first year. Values are means ±1 SE. Values followed by a different letter are significantly different (*P*<0.05).

Significant differences in the areas of wheat leaves at anthesis occurred among the treatments (Fig. 1A, B). As comparison with the non-warmed control, the percentage increase in the flag leaf area was averagely 45.7, 39.4 and 26.1% across the experimental duration in the AW, DW and NW plots, respectively (*P*<0.05). The area of green leaves at anthesis was correspondingly 25.1, 29.8 and 17.3% higher on average (*P*<0.05). Warming caused significant increases in the concentrations of flag leaf chlorophyll a and b at anthesis averagely by 3.6, 3.8 and 5.3% (*P*<0.05) in the AW, DW and NW plots, respectively (Fig. 1C). However, there was no significant difference in the rate of flag leaf photosynthesis at anthesis among the treatments (Fig. 1D).

### Aboveground plant N concentration and content

**Figure 2:**
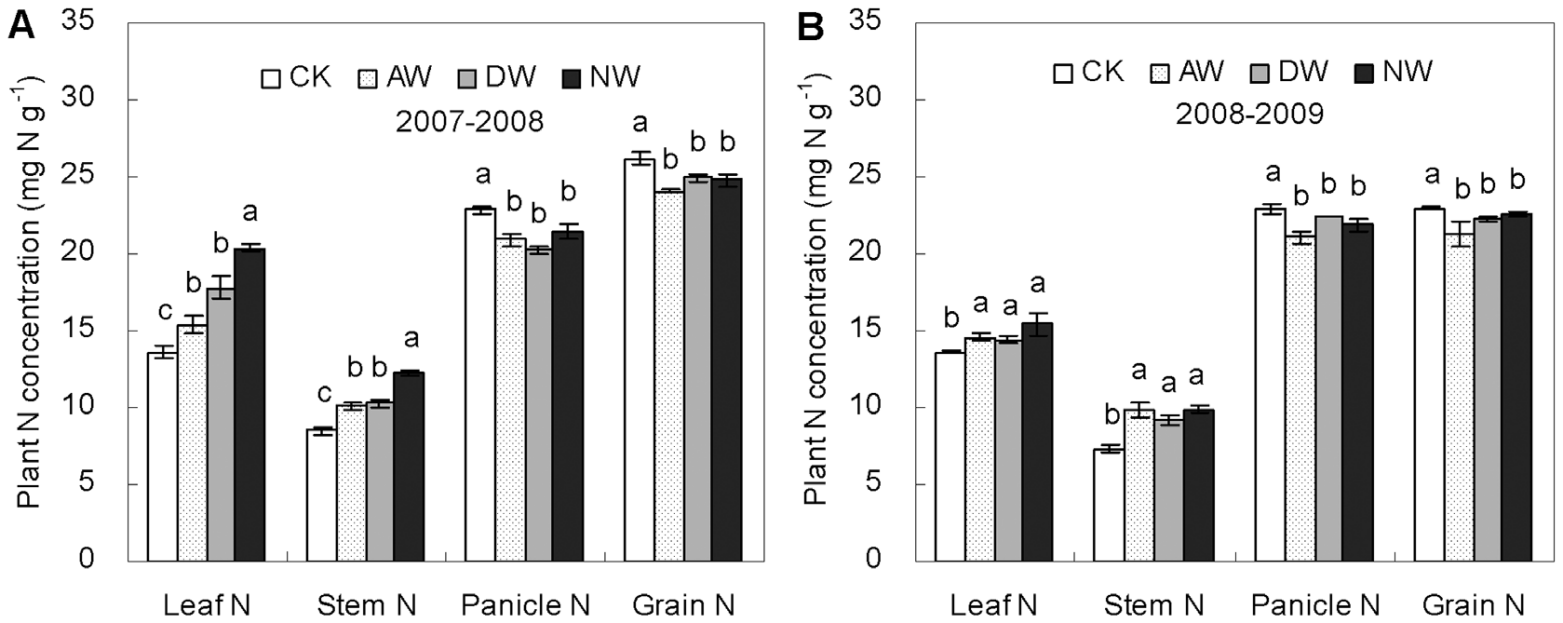
Nitrogen concentrations of leaf, stem, panicle and grain at maturity in 2007–2008 (A) and 2008–2009 (B) growing seasons in the CK, AW, DW and NW plots. Values are means ±1 SE. Values followed by a different letter are significantly different (*P*<0.05).

Warming significantly increased N concentrations in the leaf and the stem at maturity, and similar warming effects were found between the growing seasons of 2007–2008 (Fig. 2A) and 2008–2009 (Fig. 2B). The mean concentrations of leaf N at harvest were respectively 9.9, 18.3 and 31.5% higher (*P*<0.05) across the two growing seasons in the AW, DW and NW plots than the non-warmed control. For the stems, the corresponding concentrations were 26.7, 23.7 and 40.4% higher (*P*<0.05). However, warming significantly reduced panicle N concentrations (averagely by 10.2, 8.7 and 7.2%) and grain N concentrations (averagely by 9.6, 5.9 and 5.4%) in the AW, DW and NW plots, respectively.

**Figure 3:**
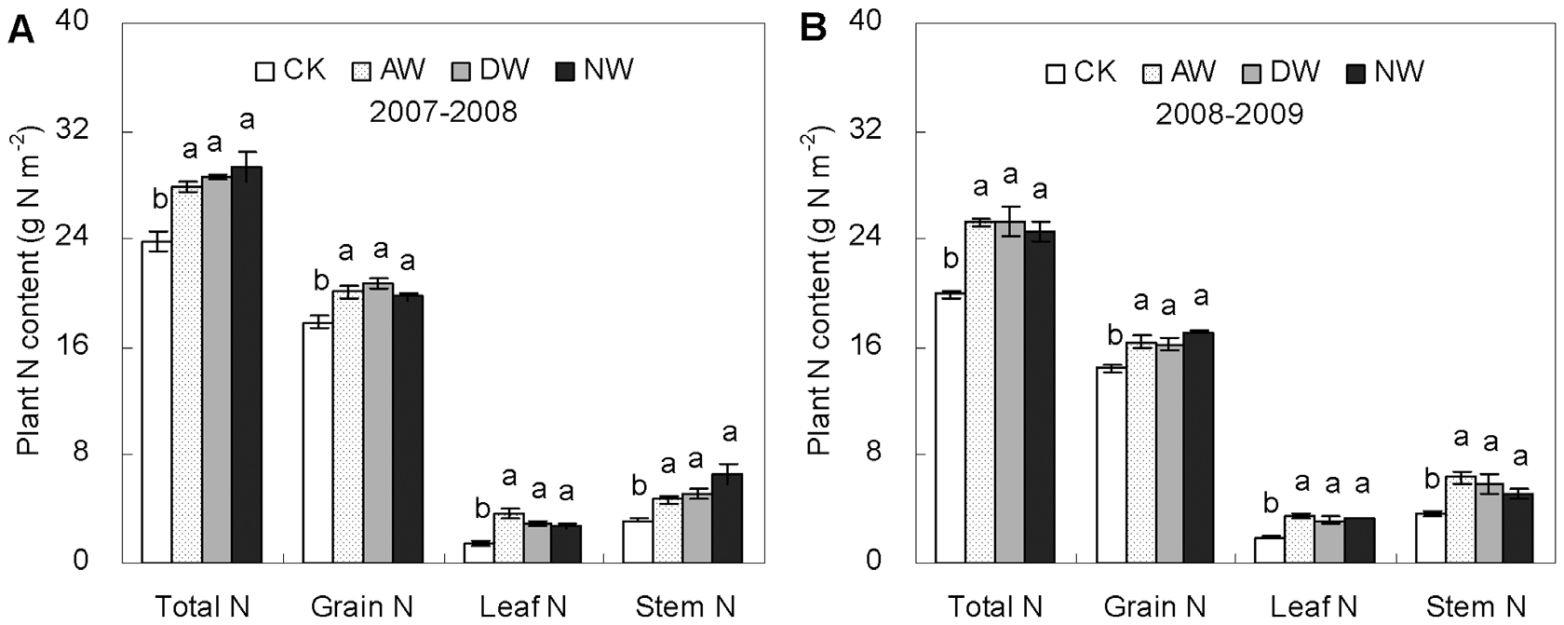
Nitrogen contents of above-ground biomass, grain, leaf and stem at maturity in 2007–2008 (A) and 2008–2009 (B) growing seasons in the CK, AW, DW and NW plots. The unit of m^−2^ is the cropland ground area. Values are means ±1 SE. Values followed by a different letter are significantly different (*P*<0.05).

Although warming impacts on plant N concentrations were different between the vegetative and reproductive parts (Fig. 2), similar warming effects on plant N contents at maturity were found between the two parts (Fig. 3). The total contents of plant N per unit area in the leaf, the stem and the grain were all increased significantly by warming (Fig. 3). At harvest, total content of aboveground plant N was averagely 30.5, 32.5 and 36.1% higher (*P*<0.05) across the two growing seasons in the AW, DW and NW plots than the non-warmed control, respectively. The increases in the content of grain N were 9.7, 14.4 and 14.2% correspondingly (*P*<0.05). Warming-led increases in the leaf N and the stem were even more (averagely by 90.1, 90.1 and 61.8% in leaves, and 50.7, 52.4 and 72.7% in stems in the AW, DW and NW plots, respectively).

### Apparent N remobilization and grain N content and weight

**Figure 4:**
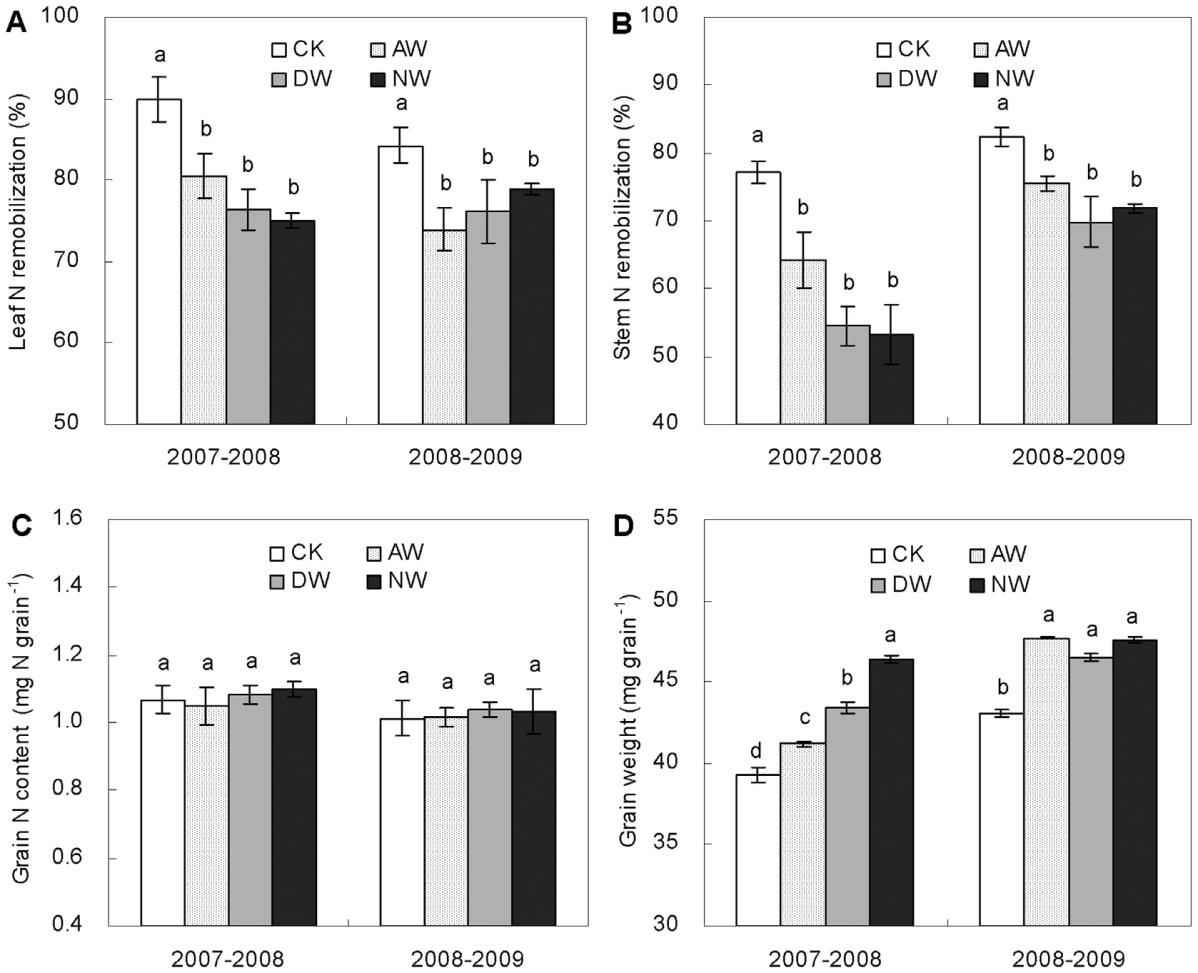
Apparent N remobilization efficiencies of leaf (A) and stem (B), grain N content *per se* (C) and grain weight *per se* (D) in 2007–2008 and 2008–2009 growing seasons in the CK, AW, DW and NW plots. Values are means ±1 SE. Values followed by a different letter are significantly different (*P*<0.05).

Warming reduced the relative content of N remobilization from the vegetative tissues to the grain (Fig. 4). The apparent remobilization efficiencies of leaf N to the grain were respectively 7.7, 12.3 and 10.3% lower (*P*<0.05) on average during the growing seasons of 2007–2008 and 2008–2009 in the AW, DW and NW plots than the control (Fig. 4A). For the stem N remobilization efficiency, warming-led decreases averaged 2.1, 15.2 and 19.1% in the AW, DW and NW plots, respectively (Fig. 4B).

There was no significant difference in grain N content *per se* between the warmed and non-warmed plots (Fig. 4C). However, grain weight *per se* was significantly higher by 6.9, 5.9 and 10.8% (*P*<0.05) on average for the two growing seasons in the AW, DW and NW plots than the non-warmed control, respectively (Fig. 4D).

### Root biomass and activity

**Figure 5:**
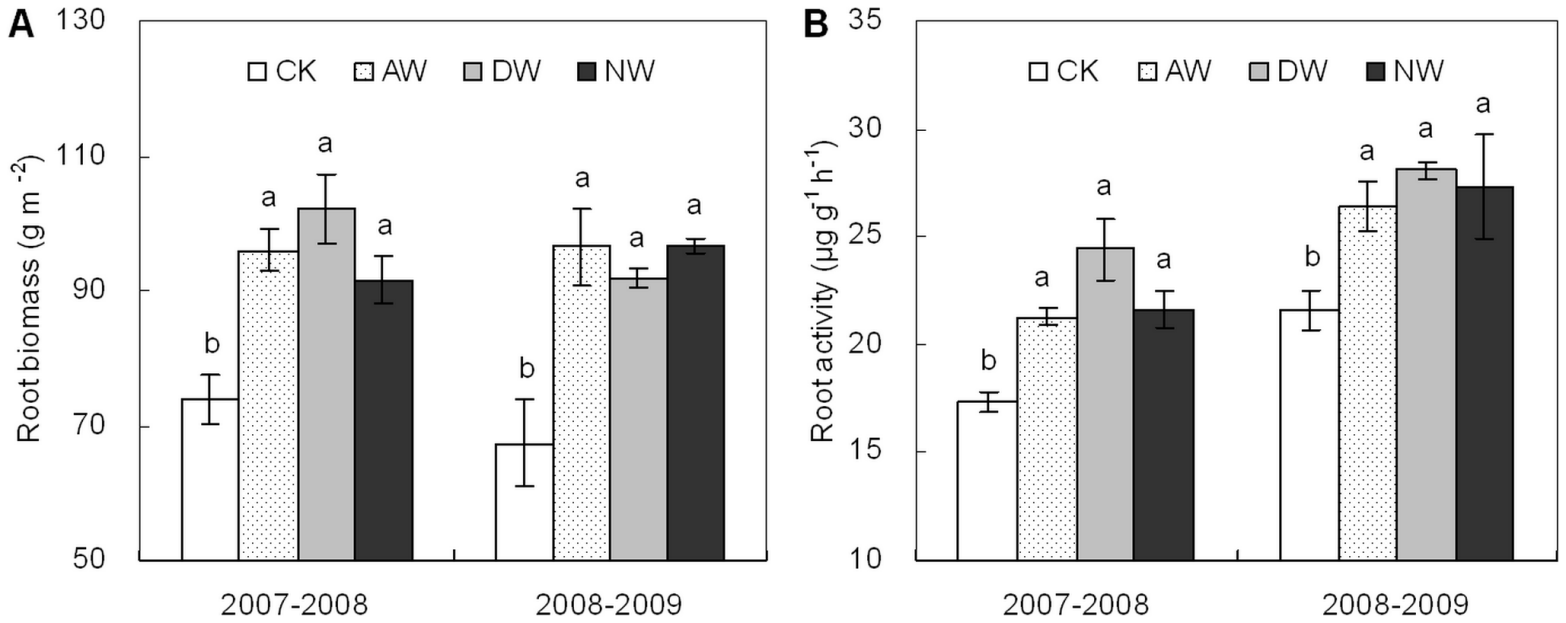
Root dry biomass (A) and activity (B) at anthesis in 2007–2008 and 2008–2009 growing seasons in the CK, AW, DW and NW plots. Values are means ±1 SE. Values followed by a different letter are significantly different within each sampling date (*P*<0.05).

Warming significantly increased winter wheat root dry biomass and activity at anthesis (Fig. 5). Root dry biomass was 36.3, 37.4 and 33.4% higher (*P*<0.05) on average across the two years in the AW, DW and NW plots than the non-warmed control (Fig. 5A). For the root activity, the corresponding increases were 22.6, 34.9 and 25.9% (*P*<0.05), respectively (Fig. 5B).

## Discussion

Warming has been predicted and experimentally shown to reduce the length of wheat growth period [11], [17], [20], [39], suggesting a large reduction in grain yield. Here, we recorded that warming by 1.5°C could significantly shorten the length of winter wheat growth period, especially the length of pre-anthesis period (Table 1). However, this moderate increase in air temperature increased wheat grain yield significantly. In fact, results of the effects of climate warming on wheat production in China have not been consistent. For example, some studies predicted that predicted warming might cause a large loss in Chinese wheat yield [8], [12], while other findings showed that climatic warming could increase crop yields in the country [21], [40], [41]. Based on warming experiment with only 100 mm irrigation, Fang *et al*. (2013) reported that warming by 2.0°C decreased wheat yield in Gucheng, North China [42]. If with an addition of 20 mm irrigation in that site, however, warming increased wheat yield significantly. With a field infrared warming facility, Hou *et al*. (2012) found that warming by 1.6°C didn't decrease wheat yield in Yucheng, Centre China, where there is no soil moisture limitation [39]. In our experiment, warming by 1.5°C at all-day, daytime and nighttime all increased wheat yield significantly in Nanjing, East China, where the natural precipitation was about 400 mm during wheat growing seasons (Fig. S2A). Together, the above evidences indicate that predicted warming will benefit irrigated winter wheat production in China.

The main reasons to the positive effects of warming on wheat production are likely attributed to the mitigation of low temperature limitation and earlier anthesis. Across winter wheat growing seasons in East China, the T_mean_ is 11.5, 8.1 and 21.7°C during the entire growth period, the pre- and post-anthesis periods, respectively (Fig. S2A). And the T_max_ often increases quickly to a high level close to 30°C at the later period of post-anthesis phase (Fig. S2A). Thus, both pre-anthesis frost/chilling and post-anthesis high temperature occur frequently, constraining winter wheat growth significantly [25], [43], [44], [45]. Warming by 1.5°C could relieve low temperature limitation, which is demonstrated by the warming-led increases in the area of green leaves and the concentration of leaf chlorophyll (Fig. 1A, B and C). Since post-anthesis photosynthesis contributes the major parts to the grain weight [46], warming-led positive effects on wheat leaves could greatly enhance biomass production and grain yield if there is no soil moisture limitation (Table 1). Meanwhile, warming-led earlier anthesis by ca. 10 d (Table 1) could advance the post-anthesis period to a lower temperature condition as compared to the non-warmed control. Consequently, no significant difference in the T_max_ was found between the warmed and non-warmed plots during the period of grain filling (Table S1). Thus, warming-led earlier anthesis might have indirectly mitigated rather than worsened the high temperature limitation to grain filling, which is partially demonstrated by the warming-led enhancement in grain weight *per se* (Fig. 4D).

The reduction in the concentration of wheat grain nitrogen (N) observed in our warming plots is puzzling, considering the fact that plants did increase N uptake from soil. Previous studies showed that post-anthesis warming could increase wheat grain N concentration due to warming-led decrease in grain starch accumulation [15], [16], [24]. However, those studies were mostly conducted for a short time with a high temperature which was above the optimum for grain filling. Thereby, existing studies might not fully present the realistic impacts of predicted warming on wheat grain N under field conditions. In our experimental site, the significantly higher N concentrations of vegetative parts (Fig. 2) and total aboveground N content at maturity (Fig. 3) indicate that warming by 1.5°C did stimulate rather than limit wheat plant N uptake. And warming-led higher root biomass and activity (Fig. 5) further showed that wheat plant N acquisition was not constrained. Thus, warming-led decrease in grain N concentration was likely due to warming-led unbalance of carbohydrate and N translocation and accumulation from vegetative parts to grains, as being demonstrated by the significant decreases in the efficiencies of apparent N remobilization in the warmed plots (Fig. 4A, B). A moderate warming significantly increased starch deposition in wheat grain (Fig. 4D), however, there was no significant increase in the content of grain N *per se* caused by warming (Fig. 4C). Thus, the lower N remobilization efficiency could be due to warming-led relative reduction in grain N deposition as compared to grain starch deposition, leading to the dilution effect observed in our experiment.

Generally, wheat grain N is mainly from pre-anthesis reserves in vegetative parts [47], while more than half of grain starch is from post-anthesis photosynthesis [48]. The rate of grain N accumulation reaches its peak prior to that of grain carbohydrate, and it declines earlier [49]. Thus, one more potential reason to warming-led decrease in grain N concentration might be due to the differential timing and sources of seed starch and protein depositions [24], [50]. In our experiment, warming significantly increased green leaf area and chlorophyll concentration (Fig. 1), suggesting that warming may have delayed the timing of leaf N remobilization. Meanwhile, warming-led positive effects on wheat leaves might maintain a long time and a large source for grain starch accumulation under a better temperature conditions (Table S1, Fig. S2A). However, direct evidence about warming impacts on the timing and sources of grain starch and protein depositions is still lacking in our study, further studies are necessary to examine the impacts of climatic warming on wheat N and carbohydrate remobilization under field conditions.

Recently, Bloom *et al*. (2010) showed that atmospheric CO_2_ enrichment reduced plant NO_3_^−^ utilization because elevated CO_2_ inhibited plant NO_3_-photoassimilation [51]. And Cheng *et al*. (2012) found that elevated CO_2_ reduced plant NO_3_^−^ uptake from soil [52]. This indicates that elevated CO_2_ may increase wheat yield but decrease grain N concentration through both the “dilution effects” [53] and decreasing vegetative N remobilization to the grain. Similarly, significant reduction was also found in wheat grain N concentration with an increase in grain yield caused by a moderate warming in our experiment. Since CO_2_ concentration and temperature are predicted to increase concurrently, future climate changes might make the problem of malnutrition more serious. Although the world's food production has increased significantly, one in twelve people worldwide are malnourished. Our study highlights the need of a mechanistic understanding of the interactive effects of elevated CO_2_ and climatic warming on plant N acquisition and translocation. The further understanding of the potential mechanisms will benefit new wheat variety breeding and cropping technique innovation for high yield with high quality under the future climate pattern.

In summary, results from our three-year experiment showed that predicted warming by ca. 1.5°C could shorten the length of wheat pre-anthesis period while the length of post-anthesis period kept almost unchanged. The grain yield was significantly higher in the warmed plots than the non-warmed control, though there were no significant differences in the biomass production among the treatments. Also, warming could increase plant N acquisition from soil; however, plants were unable to transfer this N efficiently to the grains. Consequently, warming caused significant decrease in grain N concentration while it enhanced the concentrations of leaf and stem N significantly. This is the first evidence showing that the predicted warming might enhance irrigated winter wheat productivity but decrease grain N concentration in East China. This suggests that climatic warming might intensify the problem of malnourished, hidden hunger in low-protein-intake developing countries [53].

## Supporting Information

Figure S1 **Field set-up of the experiment with the facility of Infrared Air Temperature Increase in Nanjing city, China.** This photo was taken on 10 January, 2007.
(TIF)

Figure S2 **The common daily air temperatures and precipitation averagely from 1980-2010 in the experimental site (A), the daily mean (B) and diurnal air temperatures (C) on wheat canopy and the soil moisture in 0–20 cm layer (D) under Free Air Temperature Increase (FATI) facility during the 2008–2009 growing season.** CK, AW, DW and NW are non-warmed control, all-day warming, daytime warming and nighttime warming, respectively.
(TIF)

Table S1 **Warming effects on winter wheat canopy air temperatures under the Free Air Temperature Increase (FATI) facility.**
(DOC)
