# Supplementary figures and images for: Climatic Warming Increases Winter Wheat Yield but Reduces Grain Nitrogen Concentration in East China
Source: PLoS One. 2014 Apr 15;9(4):e95108. doi: 10.1371/journal.pone.0095108 (PMC3988157; doi:10.1371/journal.pone.0095108)

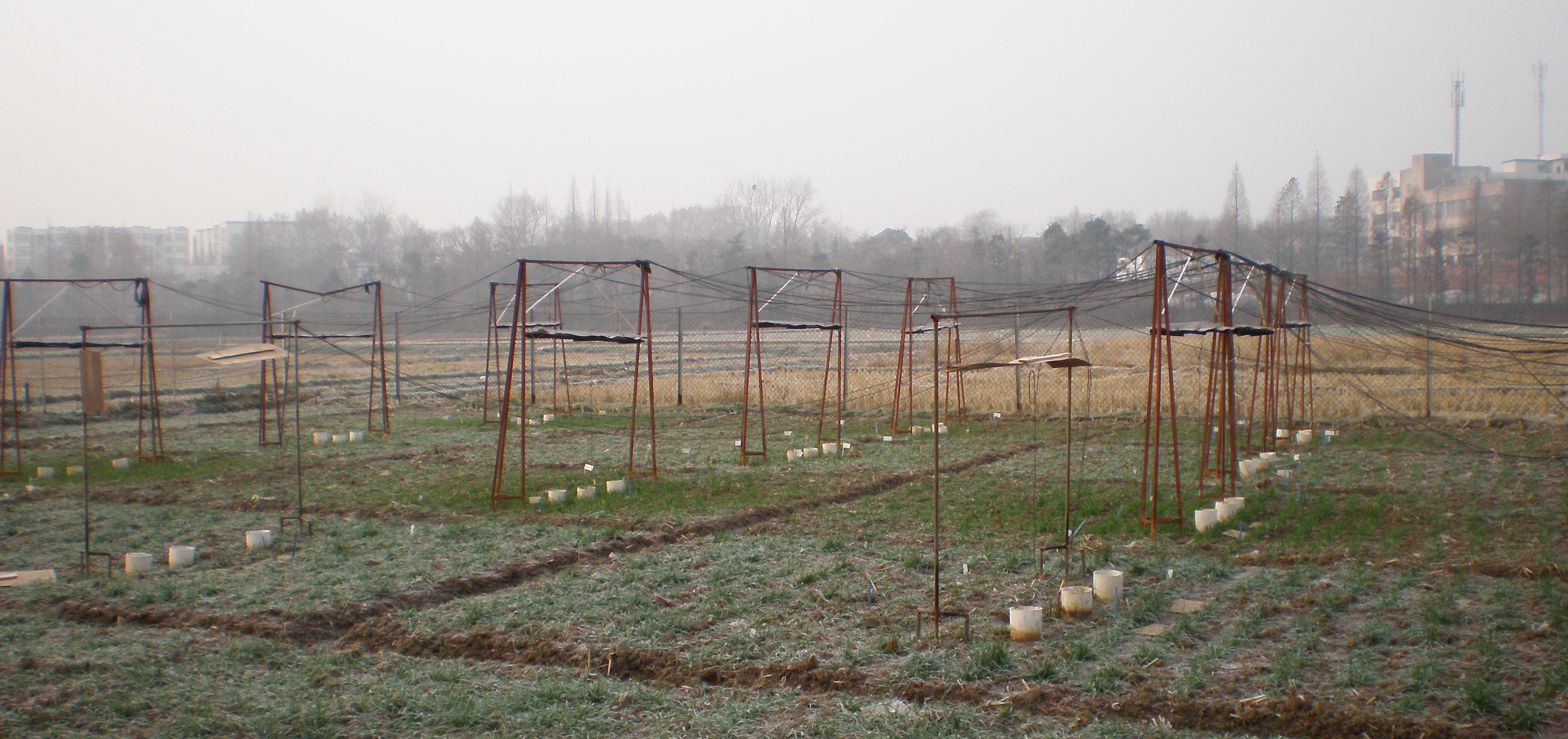

Supplement: Figure S1 — Field set-up of the experiment with the facility of Infrared Air Temperature Increase in Nanjing city, China. This photo was taken on 10 January, 2007. (TIF) [file pone.0095108.s001.tif]

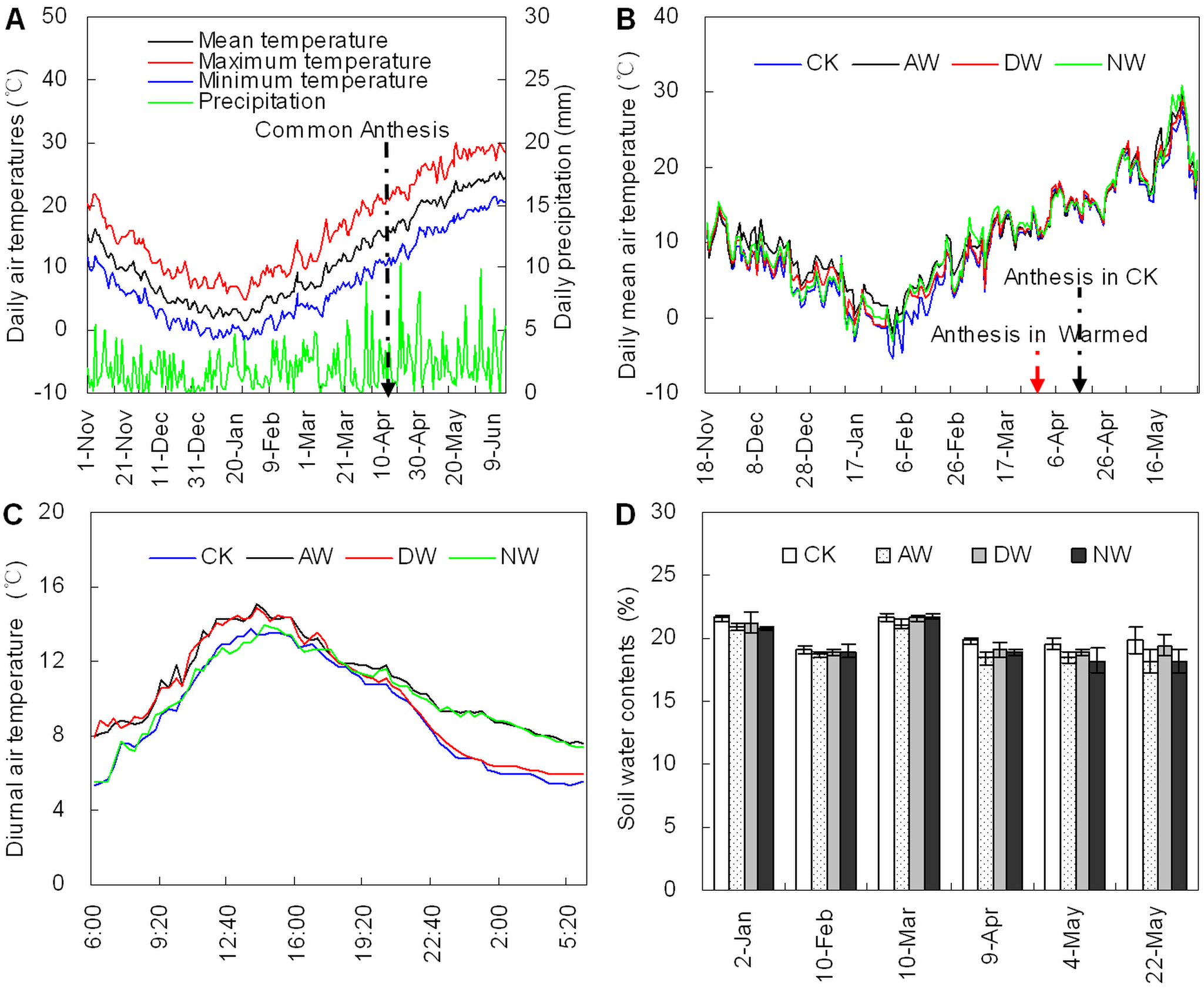

Supplement: Figure S2 — The common daily air temperatures and precipitation averagely from 1980-2010 in the experimental site (A), the daily mean (B) and diurnal air temperatures (C) on wheat canopy and the soil moisture in 0–20 cm layer (D) under Free Air Temperature Increase (FATI) facility during the 2008–2009 growing season. CK, AW, DW and NW are non-warmed control, all-day warming, daytime warming and nighttime warming, respectively. (TIF) [file pone.0095108.s002.tif]
