# Supplementary material for: Climatic Warming Increases Winter Wheat Yield but Reduces Grain Nitrogen Concentration in East China
Source: PLoS One. 2014 Apr 15;9(4):e95108. doi: 10.1371/journal.pone.0095108 (PMC3988157; doi:10.1371/journal.pone.0095108)
Supplement: Table S1 — Warming effects on winter wheat canopy air temperatures under the Free Air Temperature Increase (FATI) facility. (DOC) [file pone.0095108.s003.doc]

**Table S1. Warming effects on winter wheat canopy air temperatures under the Free Air Temperature Increase (FATI) facility.**

| Phenology | Canopy temperature | 2006-2007 | | | | 2007-2008 | | | | 2008-2009 | | | | Average over  the three years | | | |
| --- | --- | --- | --- | --- | --- | --- | --- | --- | --- | --- | --- | --- | --- | --- | --- | --- | --- |
|  |  | CK | AW | DW | NW | CK | AW | DW | NW | CK | AW | DW | NW | CK | AW | DW | NW |
| Pre-anthesis  phase (℃) | T_max_ | 12.9 | 14.3 | 14.2 | 13.3 | 19.3 | 19.9 | 20.1 | 18.9 | 15.3 | 15.9 | 15.7 | 15.3 | 15.8 | 16.7 | 16.7 | 15.8 |
|  | T_min_ | 0.7 | 1.5 | 0.6 | 2 | 3.6 | 3.2 | 3.1 | 3.9 | 0.5 | 1 | 0.8 | 0.9 | 1.6 | 1.9 | 1.5 | 2.3 |
|  | T_mean_ | 6.5 | 7.5 | 6.8 | 7.3 | 5.7 | 7.8 | 6.7 | 7 | 6 | 7.2 | 6.8 | 6.8 | 6.1 | 7.5 | 6.8 | 7 |
|  | DTR | 12.7 | 12.6 | 14.3 | 11.9 | 16.9 | 17 | 17.7 | 15.1 | 14.8 | 14.9 | 14.9 | 14.4 | 14.8 | 14.8 | 15.6 | 13.8 |
|  | AT | 928 | 931 | 931 | 916 | 1029 | 1147 | 1048 | 1072 | 979 | 927 | 963 | 938 | 979 | 1002 | 981 | 976 |
| Post-anthesis phase (℃) | T_max_ | 25.1 | 25.9 | 25.3 | 24.4 | 25.9 | 26.6 | 26.9 | 24.9 | 24.8 | 24.7 | 24.8 | 23.8 | 25.3 | 26.7 | 26.7 | 24.4 |
|  | T_min_ | 9 | 11.5 | 9.2 | 10.9 | 12.8 | 13.8 | 12 | 14.3 | 11.5 | 11.3 | 9.7 | 11.4 | 11.1 | 12.5 | 10.3 | 12.2 |
|  | T_mean_ | 17.2 | 19.1 | 18 | 18.2 | 18.4 | 19.2 | 19 | 19.2 | 17.4 | 18.9 | 18.3 | 18.8 | 17.7 | 19.1 | 18.4 | 18.7 |
|  | DTR | 15.7 | 14.5 | 16.6 | 13.3 | 14.7 | 14.6 | 16.6 | 12.3 | 13.3 | 13.4 | 15.1 | 12.4 | 14.6 | 14.5 | 16.8 | 12.7 |
|  | AT | 1079 | 1210 | 1115 | 1193 | 880 | 970 | 959 | 971 | 939 | 1149 | 1067 | 1100 | 966 | 1110 | 1047 | 1088 |
| Entire growth period (℃) | T_max_ | 17.1 | 18.3 | 18.1 | 17.2 | 19.4 | 21.2 | 21.2 | 19.1 | 17.7 | 19 | 18.6 | 18.1 | 18.1 | 19.5 | 19.3 | 18.1 |
|  | T_min_ | 3.6 | 5 | 3.6 | 5.1 | 4.3 | 5.7 | 4.2 | 6.2 | 3.5 | 4.7 | 3.7 | 4.4 | 3.8 | 5.1 | 3.8 | 5.2 |
|  | T_mean_ | 10.4 | 11.7 | 11 | 11.4 | 9.5 | 11.2 | 10.4 | 10.7 | 9.4 | 10.7 | 10.2 | 10.4 | 9.8 | 11.2 | 10.5 | 10.8 |
|  | DTR | 13.5 | 13.3 | 14.5 | 12.1 | 15.1 | 15.5 | 16.9 | 12.9 | 14.2 | 14.3 | 14.9 | 13.7 | 14.3 | 14.4 | 15.4 | 12.9 |
|  | AT | 2007 | 2141 | 2046 | 2109 | 1910 | 2117 | 2007 | 2044 | 1918 | 2076 | 2030 | 2038 | 1945 | 2111 | 2028 | 2064 |

The pre-anthesis period is from sowing to anthesis, and the post-anthesis period is from anthesis to maturity. The entire growth period is from sowing to maturity.

T_max_, T_min_, T_mean_, DTR and AT represent the average values of daily maximum temperature, daily minimum temperature, daily mean temperature, diurnal temperature range and accumulated temperature (> 0 ℃), respectively. CK, AW, DW and NW are treatments of non-warmed control, all-day warming, daytime warming and nighttime warming, respectively.
